# HPG pore: an efficient and scalable framework for nanopore sequencing data

**DOI:** 10.1186/s12859-016-0966-0

**Published:** 2016-02-27

**Authors:** Joaquin Tarraga, Asunción Gallego, Vicente Arnau, Ignacio Medina, Joaquin Dopazo

**Affiliations:** Computational Genomics Department, Centro de Investigación Príncipe Felipe (CIPF), Valencia, 46012 Spain; Departamento de Informática, ETSE, Universidad de Valencia, Valencia, Spain; HPC Service, University Information Services, University of Cambridge, Cambridge, UK; Bioinformatics of Rare Diseases (BIER), CIBER de Enfermedades Raras (CIBERER), Valencia, Spain; Functional Genomics Node, (INB) at CIPF, Valencia, 46012 Spain

## Abstract

**Background:**

The use of nanopore technologies is expected to spread in the future because they are portable and can sequence long fragments of DNA molecules without prior amplification. The first nanopore sequencer available, the MinION™ from Oxford Nanopore Technologies, is a USB-connected, portable device that allows real-time DNA analysis. In addition, other new instruments are expected to be released soon, which promise to outperform the current short-read technologies in terms of throughput. Despite the flood of data expected from this technology, the data analysis solutions currently available are only designed to manage small projects and are not scalable.

**Results:**

Here we present HPG Pore, a toolkit for exploring and analysing nanopore sequencing data. HPG Pore can run on both individual computers and in the Hadoop distributed computing framework, which allows easy scale-up to manage the large amounts of data expected to result from extensive use of nanopore technologies in the future.

**Conclusions:**

HPG Pore allows for virtually unlimited sequencing data scalability, thus guaranteeing its continued management in near future scenarios. HPG Pore is available in GitHub at http://github.com/opencb/hpg-pore.

## Background

In the beginning of 2014, Oxford Nanopore Technologies (ONT) released MinION^TM^, the first DNA sequencing device based on biological nanopores, in a limited access program that enabled researchers to use the technology for the first time. MinION^TM^ is one of the few single-molecule sequencing technologies available that produces very long reads. Moreover, MinION^TM^ constitutes the first portable high-throughput sequencer. It is the size of a smartphone and connects through a USB port to any internet-connected computer. While the technology initially produced data with a substantial amount of noise, recent practical applications have significantly demonstrated improved data quality [1, 2]. In addition, a recent report has also revealed remarkable improvements in accuracy due to enhancement of the sequencing chemistry, to the present level of 85 % for DNA reads from both strands [3]. The low cost, portability and the production of very long reads, along with a clear improvement in the quality, makes this technology one of the most promising high-throughput sequencing technologies available [4]. Nanopore sequencing has been successfully used to sequence bacterial genomes [5, 6], viral genomes [7] and eukaryotic genomes, such as yeast [8] or drosophila [9], either alone or, in combination with short read technologies [10]. Also, Nanopore sequencing technology has demonstrated its efficacy in clinics for real-time pathogen surveillance [11], because it can rapidly identify strains [12] and detect resistance genes [13], or even detecting structural variation in cancer [14].

Initially, the use of the MinION^TM^ sequencer was restricted to Windows laptops using specific cloud-based software, *metrichor*, for data handling and variant calling. The sequencer outputs binary files in the HDF5 format (http://www.hdfgroup.org/HDF5/), which once called result in 30–50 thousand binary files. However, there was no software available for accessing the data. Very recently, alternative solutions for data management and visualization have been proposed that provide more data management and visualization options and expand its use to other computer environments by using R (http://www.R-project.org/; R Core Team, 2014) [15, 16]. However, the software available was devised for managing small individual projects with a relatively low throughput, corresponding to the present-day version of the MinION^TM^ instrument. New instruments, such as the PromethION^TM^ and the GridION^TM^ are expected to be released during this year. Such devices are parallelized versions of the MinION^TM^ instrument, with an expected throughput which will overrun those of short read technologies. ONT anticipates that the current MinIon MkI will be able to generate up to 40 gigabases per run, the MinIon MkII up to 120 gigabases per run, and the PromethIon up to 6.4 terabases per run (https://goo.gl/RRPXGc). With the aim of being scalable to cope with the foreseeable increasing amounts of data generated by this technology in the near future, here we present HPG pore, a scalable toolkit for exploring and analyzing nanopore sequencing data that can run on both single computers and the Hadoop distributed computing framework.

## Implementation

### The MinION^TM^ data format

The MinION^TM^ sequencer outputs binary files in the HDF5 format (http://www.hdfgroup.org/HDF5/). The calling process generates one file for each MinION^TM^ read, which amounts between 30 and 50 thousands of individual FAST5 files (called HDF5 files with.FAST5 extension). Such files can contain a template read, and a complement read or a two-direction (2D) read (a combination of both the template and the complementary reads produced by the base-calling algorithm), alone or in any combination. The template reads are derived from the first of the two DNA strands presented to the nanopore. In the process of sequence reading, a processive motor enzyme, ligated to the leader adapter, slows down the template strands. Hairpins permit reading of the complementary strand, which produces the complement read. The change between these two sequences is recognized by the pore because an AP (apurinic/apyrimidinic) site located in the hairpin produces a specific signal. A different enzyme (named HP motor) has the mission of slowing down the complement strand. The optimal operation of the MinION^TM^ is attained when all these molecules are present and the hairpin successfully ligates both DNA strands, which then traverses the pore producing the 2D reads [2].

In addition, a FAST5 file also contains meta-information for that read and the electronic signal measured over time as a DNA molecule passes through the nanopore. A FAST5 file contains a set of hierarchical groups (with the template and complementary read), datasets and attributes (as any HDF5 file) and all the required model parameters used by the HMM for base calling. The content of FAST5 files can be visualized using the HDFView application (http://www.hdfgroup.org/products/java/hdfview/).

### Data management

HPG Pore can run both, on individual computers with a local or distributed POSIX file system such as Lustre, or on a cluster of computers by implementing the map-reduce paradigm in a Hadoop environment, the most popular open-source implementation of the map-reduce [17], a distributed programming model for processing large datasets containing relatively independent data items (http://hadoop.apache.org/). It divides data between processing nodes by splitting them into chunks (defined as key-value pairs) that are then processed separately. Users specify a map function that processes a key-value pair to generate a set of intermediate key-value pairs, and a reduce function that merges all intermediate values associated with the same intermediate key.

In the Hadoop environment, a Hadoop MapFile is used to store the individual FAST5 files into the Hadoop Distributed File System (HDFS, see http://wiki.apache.org/hadoop/HDFS). A MapFile is a sorted Hadoop SequenceFile with an index to enable lookups by using a key. A SequenceFile is a flat file containing key-value pairs within HPG Pore. Here, the FAST5 filename is stored as the key and the FAST5 file content as the value. Further, the Hadoop map-reduce framework automatically splits the MapFile into key-value pairs and calls the user map function with these pairs. The creation of the Hadoop MapFile from the FAST5 files is accomplished by executing the *import* command in the HPG Pore suite:

./hpg-pore.sh import --in/local/path/to/fast5/folder --out/path/to/hdfs/file [−−compress]

The most important command provided by HPG pore is the *stats* command to analyze and visualize the FAST5 files contents.

./hpg-pore,sh stats --in/path/to/fast5/--out/local/path/to/save/stats [−−hadoop]

To run the stats command on a Hadoop cluster, the --hadoop option is used. In this case the --in argument corresponds to the Hadoop Mapfile containing the FAST5 files, otherwise, it corresponds to the local FAST5 files folder. The --out argument indicates the folder where the results are saved: a subfolder for each run. Table 1 describes the resulting files.

**Table 1:** Files generated (for each run) by the stats command in HPG pore, where *seq* can be a template, a complement or a 2D read

Output file name	File description
summary.txt	Text file containing the number of reads and nucleotides, the mean, min. and max read length, nucleotide distribution, %GC, and mean quality
*seq*_ length_histogram.jpg	Image of the read length histogram
*seq*_content_per_pos.jpg	Image of the nucleotides (A, C, T, G, N) per position in the read
*seq*_ gc_histogram.jpg	Image of the GC histogram
*seq*_yield.jpg	Image of the number of nucleotides (yield) over time
*seq*_ quality_histogram.jpg	Image of the read quality histogram
*seq*_quality_per_pos.jpg	Image of the mean quality per position in read
*seq*_ reads_per_channel.jpg	Image of the number of reads processed per channel
*seq*_ yield_per_channel.jpg	Image of the number of nucleotides (yield) processed per channel

### Extracting plotting events as well as FastQ and FASTA files

**Fig. 1:**
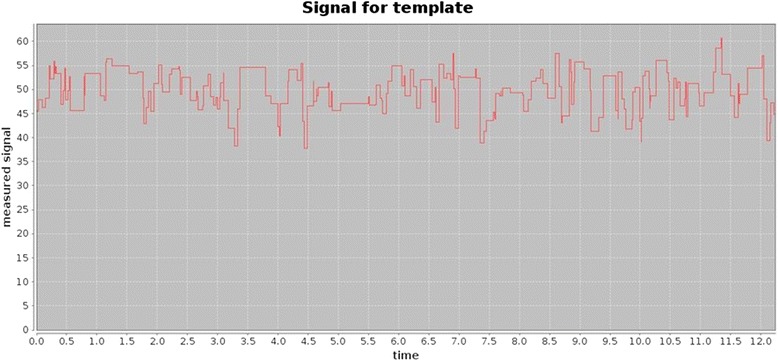
Electronic signal measured for each nanopore translocation event over time for a given MinION^TM^ template read

The *events* command extracts raw data from the electronic signal measured for a given MinION^TM^ read, and the signal command plots that signal over time (Fig. 1).

Finally, users can also extract the sequences in FastQ and FASTA formats by executing the *fastq* and *fasta* commands respectively:

./hpg-pore.sh fastq --in/path/to/fast5/--out/local/path/to/save/fastq/sequences [−−hadoop]

./hpg-pore.sh fasta --in/path/to/fast5/--out/local/path/to/save/fasta/sequences [−−hadoop]

### Availability

The HPG Pore is open source. This cross-platform software is written in Java and is available on GitHub at http://github.com/opencb/hpg-pore. A tutorial and further documentation are available at http://github.com/opencb/hpg-pore/wiki

## Results and discussion

### Features

HPG Pore has a number of features in common with the poRe and Poretools programs, but also implements several useful unique features related to quality control and other parameters of the sequence obtained, such as mean read quality, %GC, as well as plots per base sequence content and read quality histograms, among others. Some of the features that differentiate the programs originate in the different ways in which data files are managed. For instance, poRe produces one individual file for each sequence in the HDF5 file, which can cause problems with file systems quotas if a large number of reads are present in the HDF5 file. In contrast HPG Pore produces three files containing the three types of reads (template, complement and 2D), which is more convenient for further mapping with other software. Table 2 summarizes the HPG Pore features and compares them to those implemented in poRe and Poretools.

**Table 2:** Comparison of HPG Pore to the other tools available

Feature	HPG Pore	poRe	Poretools
Extract FASTq	Y	Y	Y
Extract FASTA	Y	Y	Y
Organise fast5 into run folders	–	Y	–
Create tar files of runs	–	–	Y
Organise the results into run folders	Y	–	–
Plot yield	Y	Y	Y
Plot squiggle	Y	Y	Y
Extract run stats	Y	Y	Y
Read length histogram	Y	Y	Y
read length (max., avg., min)	Y	Y	Y
Mean read quality	Y	–	–
Nucleotides content: count and %	Y	–	Y^1^
%GC	Y	–	–
Plot Frequency- %GC	Y	–	–
Plot per base sequence content	Y	–	–
Read quality histogram	Y	–	–
Reads per channel histogram	Y	Y	Y^2^
Nucleotides per channel histogram	Y	Y	–

1 Poretools does not display the nucleotide content percentage, only counts

2 Poretools returns the occupancy of pores, not the reads per channel

Like poRe and Poretools, HPG Pore produces FastQ files that can be used for downstream analysis with any conventional tool for read mapping and further variation (point mutations [12, 13] or structural variants [14]) analysis, genome assembly [13], etc. Recently appeared programs, such as NanoOK [18], provides built-in downstream analysis with an environment in which alignment can be carried out and different statistics can be obtained. However, the optimal benefit would be obtained in a near future scenario in which downstream analysis tools can natively run in the Hadoop environment. In order to avoid the transfer of HDF5 and FastQ files to a local file system, we are currently implementing read mappers, such as HPG Aligner [19], in Hadoop clusters.

### Runtimes and scalability

**Fig. 2:**
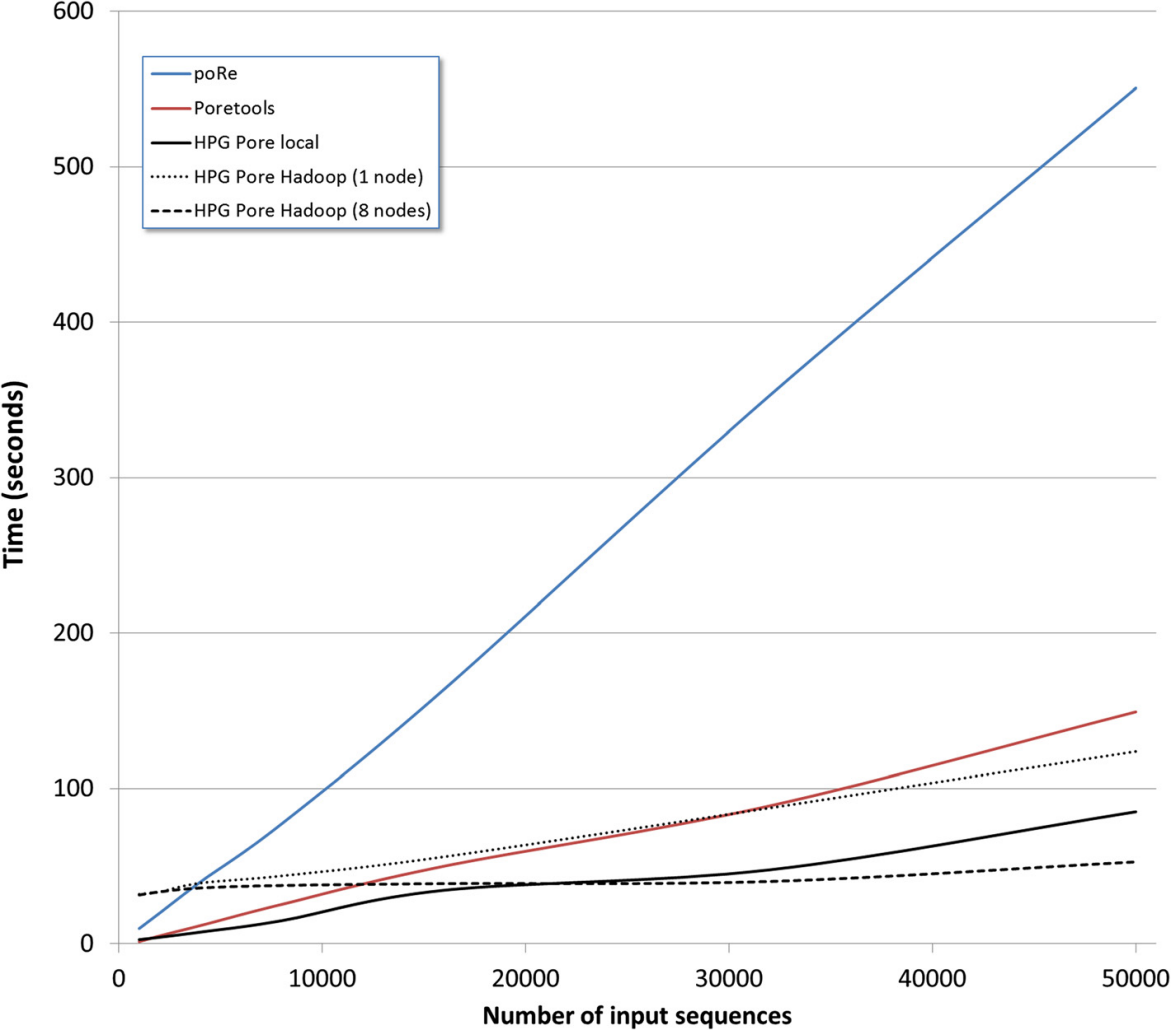
Runtimes of the three programs, poRe, Poretools, and HPG Pore, as a function of the number of sequences in the FAST5 file

Since different programs calculated different statistics, running times have been calculated for the generation of FastQ files from the original HDF5 files. The programs were ran in a Hadoop cluster with 8 nodes with 16 cores each (Intel Xeon CPU E5-2667 v2 @ 3.30GHz) and 64 GB of RAM and 12 TB distributed in 24 disks of 500GB.. We have included this information in the paper. Our study shows that runtimes in poRe, Poretools and HPG Pore (running locally) are approximately linearly dependent on the number of sequences in the FAST5 file, with a trend towards an increased slope for high numbers of sequences. HPG Pore runs the fastest, followed by Poretools, while poRe presents remarkably slower execution times (see Fig. 2). A specific problem with poRe is that the large amount of sequence files that it produces causes disk quota excess errors. To run the program with high number of reads this parameter must specifically be changed in the file system.

When HPG Pore runs in Hadoop mode it is faster than Poretools and poRe, despite an initial delay due to the preparation of the Hadoop nodes and, as expected, the speed is even faster when more nodes are available, thus it outperforms the other two programs when running in local mode (see Fig. 2). The latency of the Hadoop framework (see https://goo.gl/ujNR9F) causes the paradox that the stand alone version of HPG Pore results slightly slower than the Hadoop counterpart running on one node.

Since reads are randomly distributed across nodes in the Hadoop environment we do not expect from parameters such as read length any specific effect of runtimes or performance.

**Fig. 3:**
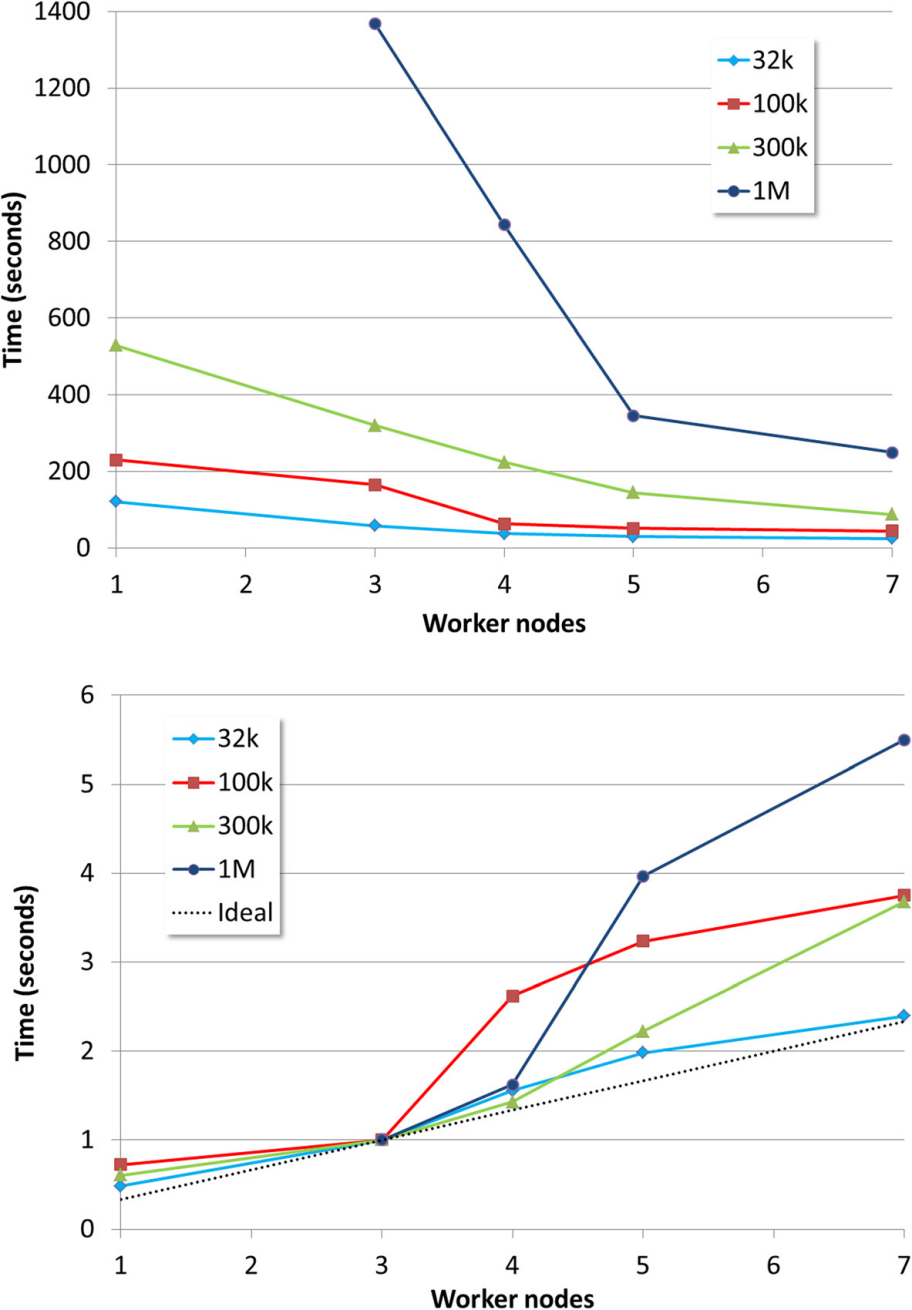
Runtimes (*upper panel*) and increase in speed (*lower panel*) as the number of nodes increase in the Hadoop system in two different scenarios: FAST5 file containing 32,000 (*blue line*), 100,000 (*red line*), 300,000 (*green line*) and 1 million (*dark blue line*) sequences. Dotted line in the lower panel represents the ideal speed-up according to the number of nodes used. Speed-ups have been calculated using 3 nodes as the starting point given that the 1 million reads could not be calculated for1 only one node

The Hadoop environment allows storage as well as speed to be scaled up. Figure 3 (upper panel) shows how runtimes decrease as the number of nodes available in the cluster increases in four different scenarios: with 32,000, 100,000, 300,000 and 1 million sequences in the FAST5 file. The speed-ups are always over the ideal expected acceleration (dotted line), and the increase in speed is clearly higher for larger data sizes (Fig. 3, lower panel).

## Conclusions

Nanopore MinION^TM^ technologies present several advantages, such as low cost, portability and the capability to produce very long reads [4] that allow anticipating an extensive use in the near future. Recently, new tools such as Poretools [16], poRe [15] and NanoOK [18] have expanded the possibilities for nanopore data management and its use in operating systems other than Microsoft. However, such programs are designed for the relatively low throughput of current nanopore devices, and even present limitations for large datasets. Moreover, the foreseeable production of enormous amounts of nanopore data by increased throughput in the future by improved versions of MinION^TM^ (the MkII version, up to 120 GB per run), as well as new nanopore instruments which have been announced (PromethIon, up to 6.4 TB per run), will soon require of scalable computational technologies to cope with these data. Here we present HPG Pore, the first scalable bioinformatic tool for exploring and analyzing nanopore sequencing data that can run both individual computers and in the Hadoop distributed computing framework. The Hadoop environment allows virtually unlimited scaling up in data size and provides better runtimes for datasets containing a large number of reads. HPG Pore allows efficient management of huge amounts of data and thus constitutes a practical solution for data analysis needs in the near future as well as a promising model for the development of new tools to deal with future genomic big data.

## Availability and requirements

**Project name:** HPG Pore

**Project home page:**http://github.com/opencb/hpg-pore

**Operating system(s):** Linux CentOS release 6.6

**Programming language:** Java

**Other requirements:** in Hadoop mode requires Hadoop installation

**License:** Apache license

**Any restrictions to use by non-academics:** no

## Abbreviations

2D: two-direction read
AP: apurinic/apyrimidinic
HDF5: hierarchical data format version 5
HP: hairpin
HPG: high performance genomic
ONT: Oxford nanopore technologies
